# Neighborhood predictors of suicide and firearm suicide in Detroit, Michigan

**DOI:** 10.1186/s40621-024-00530-4

**Published:** 2024-09-27

**Authors:** Colette Smirniotis, Veronica A. Pear, Rose M. C. Kagawa

**Affiliations:** 1 Violence Prevention Research Program, Department of Emergency Medicine, University of California, 4301 X Street, Davis, Sacramento, CA 95817 USA; 2 California Firearm Violence Research Center, 4301 X Street, Sacramento, CA 95817 USA

**Keywords:** Suicide, Firearm suicide, Youth suicide, Geographic variation

## Abstract

**Background:**

Suicide is a leading cause of death in the United States with rates increasing over the past two decades. The rate of suicide is higher in rural areas, but a greater number of people in urban areas die by suicide; understanding risk factors for suicide in this context is critically important to public health. Additionally, while many studies have focused on individual-level risk factors, few studies have identified social or structural features associated with suicide or firearm suicide, especially among young people.

**Methods:**

Study outcomes included total firearm suicide, total youth (age 10–29) firearm suicide, total suicide, and total youth suicide in Detroit, Michigan from 2012 through 2019. The predictors in this study included 58 census-tract level variables characterizing the physical features, residential stability, socioeconomic status, and demographics of neighborhoods in Detroit over the study period. We used random forest, extreme gradient boosting (XGBoost), and generalized linear mixed models to predict the four outcomes.

**Results:**

We found that the tract-level variables used in all three modeling approaches performed poorly at predicting the suicide outcomes, with area under the curve values at times exceeding 0.60 but with extremely low sensitivity (ranging from 0.05 to 0.45). However, the percentage of parcels sold in arms-length transfers in the previous 5 years, the count of vacant lots per square mile, and the percentage of children aged three and older who were enrolled in preschool each demonstrated associations with at least two of the outcomes studied.

**Conclusions:**

Our findings suggest place-based factors at the tract level do not provide meaningful insight into the risk of suicide or firearm suicide among youth or the general population in Detroit, Michigan. Future practice and study should consider focusing on both larger and smaller areas, including city and individual-level factors. For example, studies might benefit from the use of both neighborhood and individual-level measures and their interactions to improve our understanding of place-based risk factors and suicide risk.

## Background

Suicide is a leading cause of death in the United States (Centers for Disease Control and Prevention 2022). While the highest rates of suicide are among males over the age of 85 (57.8 per 100,000), the years of potential life lost due to suicide are the highest among males in their 20s. For young people aged 10–29, suicide is the second leading cause of death, preceded only by unintentional injury (Centers for Disease Control and Prevention 2022). For youth and young adults racialized as Black, rates of suicide have increased dramatically (by 36.6%) since 2018 (Stone 2023). These alarming trends amplify the need for further research. Additionally, while rates of suicide are highest in rural areas, far more suicide deaths occur in urban areas due to the concentration of people in cities and metro areas (Centers for Disease Control and Prevention 2022). Understanding risk factors for youth and young adult suicide in urban populations is of critical importance.

Firearms are the most common method of suicide (Centers for Disease Control and Prevention 2022), at least in part due to their lethality; it is estimated that 90% of suicide attempts with a firearm result in death (Conner et al. 2019a). Firearm availability is also associated with increased firearm suicide risk in United States cities (Miller et al. 2013). Additionally, firearm suicide deaths are more likely to occur in areas with high rates of firearm availability (Kaplan et al. 2009).

Research on suicide and firearm suicide tends to focus on individual-level risk factors such as mental illness and mood disorders, substance use disorders, medical conditions, firearm purchases and means availability, and relationship challenges (Boggs et al. 2018; Schleimer et al. 2023, 2021; Conner et al. 2019b; Bilsen 2018). There is far less research on the social or structural factors that influence suicide risk specifically, with a few notable exceptions. Economic contraction and financial strain, which are associated with longer periods of unemployment duration and housing loss, are associated with increased suicide risk (Fowler et al. 2015; Classen and Dunn 2012). These economic factors likely exacerbate the influence of individual level risk factors such as risky alcohol consumption (Kaplan et al. 2015). A systematic review of urban contextual factors and suicide risk concluded urban areas with higher levels of socioeconomic deprivation and social fragmentation were at elevated risk for self-harm and suicidality (Satherley et al. 2022). As one additional example, the availability and quality of the mental health care service network is also critically important for suicide risk (Lang 2013; Stone et al. 2017).

A larger literature has focused on how the built environment and social and economic context might shape the mental health experience of individuals, which is in turn related to suicide risk. The material infrastructure of neighborhoods, the social connectedness of their residents, and economic opportunity can influence psycho-social stress and mental health (Clark et al. 2007; Latkin and Curry 2003). Additionally, neighborhoods with more “problems” such as violence, heavy traffic, and lack of parks or playgrounds, tend to have higher rates of depression, even after controlling for individual-level factors (Echeverría et al. 2008). In a county-level study in the United States, ecological measures of the food environment and access to exercise were associated with fewer average poor mental health days while violent crime and income inequality were associated with more average poor mental health days (Olson-Williams et al. 2023).

Social norms, economic stability, and physical features of the environment are associated with mental health and well-being (Mukherjee et al. 2021). Yet little research has focused on the role of these contextual factors in shaping suicide risk in urban areas. Additionally, the ways in which these factors influence suicide risk may differ across localities and populations. The current study takes place in Detroit, Michigan, a city with a majority Black population (greater than 75%) and a specific set of place-based, social, and economic features (United States Census Bureau: Dicennial Census 2010, 2020). The median household income in Detroit in 2019 was slightly above $30,000, and approximately one-third of the population in Detroit live below the poverty level (United States Census Bureau 2019)—figures that are far beyond the national average. Crime rates in Detroit are also among the highest in the nation, with approximately 1965 violent crimes and 4303 property crimes per 100,000 residents (US Department of Justice, Federal Bureau of Investigation 2019).

Given the alarming rise in the of rate of suicide among Black youth and young adults, and the need to better understand mental health and suicide risk in urban contexts (Núñez-González et al. 2020), this study aimed to identify features of the social and structural environment that are predictive of suicide risk and may point to interventions with potential for wide-ranging benefits. We hypothesized that higher levels of neighborhood instability, indicating lower levels of social cohesion, and greater socioeconomic deprivation would be most predictive of suicide risk.

## Data

Our study population included all neighborhoods in Detroit, Michigan over the study period 2011–2019, with outcomes measured 2012-2019 and predictive features measured 2011–2018. The primary unit of analysis was census tracts (N = 297 based on 2010 Census tracts). We used census tracts as our primary unit with the assumption that the people, physical environment, and economic conditions of census tracts are salient contextual features of the neighborhood. However, the true boundaries of neighborhoods recognizable to the people who reside in them are difficult to ascertain (Coulton et al. 2013).

The outcomes of interest included the count of firearm suicides (International Classification of Diseases (ICD) codes X73-X75), youth firearm suicides (age 10–29), all suicides (ICD codes X60-X84), and all youth suicides (age 10–29), obtained from the Michigan Department of Health and Human Services (World Health Organization 2004; Michigan Department of Health and Human Services 2019). We use the term “youth” to include both children and young adults; youth suicide is a clear concern, and, as previously noted, rates of suicide are particularly high among 20–29 year old young adults. These data were geolocated by decedent place of residence and aggregated to census tracts. Because suicide is a rare event in statistical terms, we converted the counts to binary outcomes indicating the presence or absence of each outcome in each tract-year. All covariates were lagged one year with respect to the outcome. For example, firearm suicides occurring in 2012 were predicted using covariates that described the state of census tracts in 2011.

The fifty-eight predictor variables (including year) can be grouped into four main categories: physical features, residential stability, socioeconomic features, and demographic features. We also included the latitude and longitude of each census tract’s centroid to capture some element of the spatial relationship between tracts (Li 2022). Physical features include variables such as road density and walkability; the count of schools, alcohol outlets, and religious buildings; and percent of land that is protected as well as counts of single family homes per square mile, commercial buildings per square mile, historical redlining grade, and the count of buildings with quality grade D or F (Data Driven Detroit 2009; Dynamo Metrics 2020; United States Environmental Protection Agency 2018; National Center for Education Statistics; Centers for Medicare and Medicaid Services; United States Department of Homeland Security, Geospatial Management Office; Esposito et al. 2020; Finlay et al. 2020; United States Environmental Protection Agency 2020; Nelson et al. 2020). The counts of hospitals and federally qualified health centers are included as physical features in an attempt to represent the availability of mental health care. Measures of residential stability include the percentage of housing units that are renter-occupied, recent movers (scaled by GeoLytics from 0 to 1000, where 100 is the national average), the percentage of parcels with arms-length transfers in the previous 5 years, and the maximum number of arms-length transfers for a parcel in the tract (Geolytics Inc. 2019; Dynamo Metrics 2020). An arms-length transfer is a real estate transaction between two independent parties who do not have an existing relationship. Here, arms-length transfers serve as a proxy for a home being in a cycle of high turnover and thus indicative of lower social cohesion. Socioeconomic features include the percentage of the census tract population that was unemployed, the percentage of children enrolled in private schools, and median household income. The percentage of tract residents that were naturalized citizens and racial and ethnic categories are among the demographic features included (Geolytics Inc). We also included two measures of economic and racial segregation calculated based on the Index of Concentrations at the Extremes (ICE), which previous studies have found to be associated with fatal and non-fatal assaults (Krieger et al. 2017). To measure residential economic segregation, the metric is calculated as $$ICE_i=\frac{(A_i-P_i)}{T_i}$$, where $$A_i$$ is the number of high-income earners, $$P_i$$ is the number of low-income earners, and $$T_i$$ is the total count of people with income data in tract *i*. The 20th and 80th U.S. household income percentiles were used as the high- and low-income thresholds (Krieger et al. 2017), which were $25,000 and $100,000 in 2010 (United States Census Bureau 2020). To use ICE as a measure of residential racial segregation, the same formula is used but with $$A_i$$ as the number of non-Hispanic White residents, $$P_i$$ as the number of non-Hispanic Black residents, and $$T_i$$ as the total count of people with race and ethnicity data in the tract *i*. A complete list of variables can be found in Appendix B.

There were missing values present in three variables. Alcohol outlet data was available for 2010 through 2015 and 2017 only. We used modified interpolation for 2016 and carried forward 2017 values for 2018 and 2019. Missing values occurred in two other variables: the count of poor quality buildings per square mile (15.03% missing) and historical redlining grade (7.74% missing). Because some of our methods did not allow for missing values, we imputed them using random forest proximity (Stekhoven and Buehlmann 2012; Stekhoven 2022).

Prior to modeling, we identified and removed highly correlated variables (defined as an absolute value of correlation greater than 0.6) within each of the four categories of variables described above (physical features, residential stability, socioeconomic features, and demographic features). Among the demographic variables, ICE-race was highly correlated with the racial and ethnic categories (percentage Black, Hispanic, White, and other), the percentage of residents that were naturalized citizens, and the percentage of residents that were not citizens. We removed the variables with which ICE-race was highly correlated and retained ICE-race, population, and the percentage of the population that was 15–29 year old males. In the residential stability category, recent movers and the percentage of residents that were renters were highly correlated; we excluded recent movers. In the socioeconomic features category, ICE-income was highly correlated with median household income and the percentage of the population below 150% of the poverty line, and the percentage of parcels that were tax foreclosed was highly correlated with the percentage of parcels that were tax delinquent; we removed the median household income, percentage below poverty, and percentage tax foreclosed measures. Of the physical feature variables only the acres of land protected from development and the percentage of land protected from development were highly correlated; we excluded the amount in acres of protected land. In total, we removed 11 variables in this process, leaving 47 covariates for use in all models.

For XGBoost, all predictor variables were normalized to generate 0–1 data, and for the regression models, all predictor variables were standardized to facilitate interpretation. We attempted the use of non-standardized variables, but these models did not converge.

## Methods

We began by exploring the data descriptively. We used measures of central tendency such as means and percentages to compare tract-years with and without firearm suicides and youth firearm suicides, excluding missing values. To assess significant differences, we estimated a series of generalized linear mixed effects logistic regression models (GLMMs)–modeling each covariate on its own, controlling for population and a random intercept for census tract and using a 0.05 significance level. We repeated this process for each of the outcomes.

For each year in the study period, we also calculated a Moran’s I statistic for each outcome to assess the presence of spatial autocorrelation.

We used machine learning algorithms and mixed effects logistic regression to develop prediction algorithms. Predictive accuracy was assessed for all approaches through performance metrics, including area under the ROC curve (AUC), sensitivity, balanced accuracy, and area under the precision-recall curve (AUPRC), a metric often used for imbalanced data. For the machine learning algorithms, we partitioned our data into training and test sets, such that 80% of census tracts were included in the training set and the remaining 20% were used in the test set.

We implemented random forest, which performs well in a variety of settings, including rare event detection (Muchlinski et al. 2016). Briefly, random forest is an ensemble of decision trees, each built from a random bootstrap sample of data. Each tree uses a recursive process of splits, built with a random sample of *mtry* variables at each node, with the best one picked to partition the data into two at each node (where “best” means it splits the data into the two most homogeneous groups in the classification setting). This process is repeated until node purity or a minimum node size is reached. Each tree in the forest classifies each observation, and the random forest aggregates across the tree votes (Breiman 2001; Hastie et al. 2009). Individual trees are prone to overfitting, producing low bias but high variance estimates. By aggregating across all trees, the random forest algorithm reduces this variance (Breiman 2001; Hastie et al. 2009). Because each tree consists of only a sample of the data, it uses an out-of-bag error estimate that is equivalent to N-fold cross validation error as the size of the forest increases, making it unnecessary to also implement cross validation when using random forest (Hastie et al. 2009). Additionally, random forest allows for undersampling as a way to address imbalanced data. When creating the bootstrapped sample to build each tree, it can select a stratified sample. This ensures each tree is built using balanced data, and its performance can still be tested with imbalanced data. We employed a stratified random forest on the training data, using the case count in the training set as the strata size, tuning the *mtry* parameter to maximize area under the ROC curve (AUC).

We also employed extreme gradient boosting, or XGBoost, another ensemble machine learning algorithm. Boosting is a method that recursively combines models together, with each model predicting the residuals of the prior one. It minimizes a regularized objective that consists of a loss function and a complexity penalty using gradient descent. This process continues until some minimum threshold in prediction improvement is met (Chen and Guestrin 2016). We assigned weights to the minority class that were equal to the ratio of the majority class size to the minority class size and kept weights for the majority class at 1. We used 5000 rounds of five-fold cross validation with XGBoost and tuned the hyperparameters using a random search with 50 iterations, with value ranges shown in the first 6 rows of Table 3. With those parameter values, we then tuned the number of rounds as shown in the last row of Table 3. For all tuning purposes, the evaluation metric was AUC to account for the imbalanced data set. We assessed performance on the test set using the metrics described above. All tuning and building was done using R packages xgboost and mlr (Chen et al. 2024; Bischl et al. 2016; Lang et al. 2014; Bischl et al. 2016, 2017; Probst et al. 2017; Casalicchio et al. 2017).

Finally, we estimated a series of generalized linear mixed effects logistic regression models with a random intercept for census tract to identify risk factors for each outcome. The variables in these models are standardized with mean of 0 and standard deviation of 1 to facilitate interpretation. We first used all of the de-correlated variables as independent predictors (denoted Model 1). We then ran a model including only the significant predictors ($p<0.10$) from the previous model (denoted Model 2). We repeated this process using population as an offset instead of a covariate, first with all de-correlated variables (denoted Model 3) and then with only significant covariates from Model 3 (denoted Model 4). For the models with an offset, we dropped three census tracts with zero population from the analysis. These models were built using R package glmmTMB (Brooks et al. 2017). To distinguish between outcomes in this paper, an abbreviation is attached to each model number: “FS” for firearm suicide, “YFS” for youth firearm suicide, “S” for all suicide, and “YS” for all youth suicide.

## Results

In the next section, we show results of our exploratory analysis and then separately show machine learning and model results for each of the four outcomes. We present detailed results corresponding to the analysis for the firearm suicide outcome in the text below, while tables for the additional outcomes can be found in the appendices. Information on tuning parameters for random forest and XGBoost can also be found in the Appendix (Tables 3 and 4).

### Exploratory results

Summaries of all covariates from all census tracts for the duration of our study period can be found in Table 5. Among all tracts, there were 203 tract-years with at least 1 firearm suicide and 2173 tract-years with 0 firearm suicides. With the three zero-population census tracts removed, there were 2149 tract-years with 0 firearm suicides, while the firearm suicide count remained the same. Comparing the values in Table 5, tract-years with firearm suicide deaths tended to have more condominiums per square mile, a higher average percentage of parcels with arms-length transfers in the last 5 years, and fewer vacant lots per square mile. (See also Table 1.)

There were 61 tract-years with youth firearm suicide, and there were no covariates that demonstrated a statistically significant association with the outcome in their individual GLMMs (Table 6).

There were 348 tract-years with any suicide, and four covariates showed significant differences between the tracts with and without suicide (Table 1). Tracts with suicide had a higher average maximum number of arms-length transfers per parcel, a higher average percentage of parcels with an arms-length transfer, a higher average percentage of children aged three and older in preschool, and a lower average number of vacant lots per square mile than tracts without suicide.

There were 113 tract-years with youth suicide, and two covariates with significant differences; tracts with youth suicide showed a higher percentage of parcels with an arms-length transfer and a higher percentage of children aged three and older in preschool than tracts without youth suicide (Table 1).

Using Moran’s I test, we did not detect spatial autocorrelation in the majority of years, with the exception of counts of firearm suicide in 2012 and youth firearm suicide in 2014 (Table 7).

**Table 1:** Select census tract features by presence of suicide outcome

Outcome	Variable	Tract-Years w/o Outcome		Tract-Years w Outcome	
		Median	Mean (SD)	Median	Mean (SD)
FAS	% parcels w arms-length transfer (5 yrs)	0.03	0.03 (0.03)	0.03	0.04 (0.03)
FAS	Condos per sq. mi	1.96	14.74 (39.90)	2.27	22.96 (56.96)
FAS	Vacant lots per sq. mi	775.38	948.21 (719.90)	583.95	764.71 (610.97)
S	Max # of arms-length transfers per parcel	2.00	2.04 (1.04)	2.00	2.34 (1.06)
S	% parcels w arms-length transfer (5 yrs)	0.02	0.03 (0.03)	0.04	0.04 (0.03)
S	% children aged 3+ in preschool	1.89	2.01 (1.07)	2.14	2.16 (1.06)
S	Vacant lots per square mile	785.61	961.43 (729.87)	643.96	764.09 (578.04)
YS	% parcels w arms-length transfer (5 years)	0.03	0.03 (0.03)	0.04	0.04 (0.03)
YS	% children aged 3+ in preschool	1.91	2.02 (1.07)	2.21	2.25 (0.98)

Summary statistics comparing tract-years with and without given outcome for covariates that were significant at the 0.05 level in a generalized linear mixed effects logistic regression with a random intercept by census tract using only the single covariate and controlling for population

### Firearm suicide

Both machine learning algorithms demonstrated poor prediction performance for firearm suicide. Using the test set, the predictions from the stratified random forest yielded sensitivity of 0.10, AUC of 0.54, and AUPRC of 0.09 (Table 2). XGBoost showed similar performance to random forest: sensitivity of 0.05, AUC of 0.59, and AUPRC of 0.10. All model performance metrics are shown in Table 2.

**Table 2:** Model metrics: Firearm suicide

Model	AIC	AUC	Sens	Spec	AUPRC	Balanced Accur
Random Forest	–	0.5423	0.1000	0.8955	0.0921	0.4977
XGBoost	–	0.5887	0.0488	0.9317	0.1043	0.4902
Model 1, all vars	1427.1	0.6536	0	1	0.1512	0.5000
Model 2, selected vars	1378.8	0.6105	0	1	0.1217	0.5000
Model 3, offset, all vars	1419.2	0.6556	0	1	0.1522	0.5000
Model 4, offset, selected vars	1369.0	0.6224	0	1	0.1402	0.5000

Model 1-FS used all de-correlated variables as predictors. In Model 2-FS, we used only the significant (at $\alpha =0.10$) covariates from Model 1-FS: latitude, large multi-family units per square mile, vacant lots per square mile, count of hospitals, count of religious buildings, percent of children aged 3+ in preschool, and historical redlining grade. Model 3-FS used all de-correlated variables except population as a predictor, instead including population as an offset (where population remained on its original scale). Model 4-FS used the significant covariates from Model 3-FS with a population offset: small multi-family units per square mile, large multi-family units per square mile, vacant lots per square mile, percent of parcels with vacant buildings, count of hospitals, percent of housing occupied by renters, percent of children aged 3+ in preschool, and historical redlining grade. All four models also do a poor job of predicting firearm suicide tract-years (Table 2); predictions from these models are all zero (i.e. predict no firearm suicide in all tract-years).

### Youth firearm suicide

The predictions of youth firearm suicide from the stratified random forest yielded a sensitivity of 0.25, AUC of 0.67, and AUPRC of 0.05 (Appendix Table 8). XGBoost showed worse performance than random forest: sensitivity of 0.17, AUC of 0.43, and AUPRC of 0.024.

For youth firearm suicide, Model 2-YFS and Model 4-YFS included the only significant variables from their full model counterparts: industrial buildings per square mile and road network density, plus population (as covariate in Model 2-YFS and offset in Model 4-YFS). The performance of these models was poor, with no predictions of the minority class (Table 8).

### Suicide

The predictions of suicide from the stratified random forest displayed a sensitivity of 0.25, AUC of 0.59, and AUPRC of 0.19 (Table 9). XGBoost here performed similarly to random forest: sensitivity of 0.26, AUC of 0.53, and AUPRC of 0.20.

Model 2-S used the covariates vacant lots per square mile, percent of parcels undergoing arms-length transfer in previous 5 years, percent of children aged 3 and older enrolled in preschool, historic redlining grade, racial residential segregation, and population. Model 4-S included vacant lots per square mile, percent of parcels with vacant buildings, percent of parcels undergoing an arms-length transfer in previous 5 years, percent of children aged 3 and older enrolled in preschool, historic redlining grade, and population offset. These models performed poorly, with no predictions of the minority class (Table 9).

### Youth suicide

The predictions of youth suicide from the stratified random forest displayed a sensitivity of 0.45, AUC of 0.69, and AUPRC of 0.09. XGBoost performed slightly worse than random forest: sensitivity of 0.09, AUC of 0.61, and AUPRC of 0.06. More performance metrics for these algorithms are shown in Table 10.

For all youth suicide, Model 2-YS used the covariates industrial buildings per square mile, percent of parcels with vacant buildings, percent of renter-occupied housing units, percent of children aged 3 and older enrolled in preschool, percent of tax-delinquent parcels, racial residential segregation, and population. Model 4-YS was similar, excluding racial segregation and instead including population as an offset. These models performed poorly, with no predictions of the minority class (Table 10).

## Discussion

This study aimed to identify the physical, social, and economic features of the neighborhood environment that contribute most to the prediction of suicide and firearm suicide overall and among youth and young adults in Detroit, Michigan from 2012 to 2019. Identifying features of the social and structural environment that are predictive of suicide risk may point to interventions with potential for wide-ranging benefits. Using the machine learning algorithms random forest and XGBoost, as well as mixed effects logistic regression, we found that the variables we used were unable to predict firearm suicide well at the census tract level. Given that machine learning algorithms like XGBoost and random forest typically perform well with rare events, especially compared to traditional models, our results suggest the included variables provide little to no predictive insight at this level of granularity in our study site.

While model performance was poor overall, there were a few patterns that emerged from this analysis. The percentage of parcels sold in arms-length transfers in the previous 5 years exhibit a significant positive association in exploratory analyses with three outcomes (firearm suicide, suicide, and youth suicide), as well as appearing statistically significant in both full GLMMs for the suicide outcome (Models 1-S and 3-S). A higher percentage of arms-length transfers can indicate higher neighborhood turnover and potentially lower social cohesion. Several other studies have found a relationship between social isolation and adverse mental health effects (Fedina et al. 2023; Lim et al. 2017). The count of vacant lots per square mile showed a negative association with firearm suicide and suicide, and it also appeared as significant in Models 1 and 3 for firearm suicide and suicide. We hypothesize that the count of vacant lots per square mile may simply be a proxy for population at risk that is not otherwise captured by our variables: fewer vacant lots may indicate greater population density. Finally, the percentage of children aged three and older enrolled in preschool showed a significant positive association with suicide and youth suicide; it was also significant in Models 1 and 3 for firearm suicide, suicide, and youth suicide. This finding is unexpected, and we are unaware of a plausible explanation for this relationship.

Differences in our findings and those of some prior studies may in part be explained by our relatively unique study population. Whereas other studies that used statewide data found that non-metropolitan areas had higher suicide risks than metropolitan areas and that predominantly Black neighborhoods had lower suicide risk than neighborhoods that were not predominantly Black, Detroit as a city has relatively low variation within its boundaries with respect to race, urbanicity, and economics (Sugg et al. 2023; Fontanella et al. 2018). We posit that the lack of variation in citywide data compared to statewide data may account for our different findings. It is possible we may have detected associations if we were making comparisons across a more diverse area with respect to those three variables.

By limiting the study to neighborhoods in Detroit, we are looking at a restricted area of the full distribution of the studied variables; socioeconomic features in much of the city of Detroit tend to indicate reduced wealth and lower social and economic opportunity. Perhaps in this context, neighborhood features are less important than city features, and policies or programs that improve conditions for the city as a whole would be of greatest benefit. Additionally, this study did not include individual-level data and as such, cannot shed light on individual risk factors, though previous research indicates that programs tailored to individuals at greater risk are a critical element of prevention (Stone et al. 2017).

As a predominantly Black metropolis, the city’s population overall is at lower risk for suicide death than among other groups, especially when compared to a statewide population. However, it is important to emphasize that the suicide rate among Black Americans has increased significantly in recent years (Stone 2023). This coincides with an increase in Black gun ownership. During the 2020 surge in gun purchasing, Black individuals were 1.7 times more likely than white individuals to become first time gun owners (Simonson et al. 2021). These are concerning trends that deserve more attention and further study. Given how recently firearm ownership trends have changed among Black individuals, future studies are needed that also include death data from after 2020.

In addition to adding to the body of work about suicide in a primarily Black city, this study also contributes further research on the role of environmental factors and suicides and firearm suicides, particularly at the census tract level. Given that previous studies have shown individual-level factors are important but found mixed results for contextual variables measured at the area level, we hypothesized that analysis at the census tract-level, a relatively small areal unit, could provide useful insights (Rehkopf and Buka 2006). This study considers a large number of variables, including commonly used demographic and socioeconomic variables as well as the somewhat novel addition of building and land information; this better captures the neighborhood environments in which people live compared to previous studies with fewer such variables. Finally, it utilizes both traditional regression modeling techniques and novel machine learning algorithms, which handle rare outcomes and collinearity well and do not necessitate distributional assumptions.

This study should be interpreted with consideration of the following limitations. First, our proxies for mental health care availability (hospitals and federally qualified health centers) do not fully capture the range of mental health services available in Detroit; more explicit measures of mental health care availability (e.g. provider density) were found to be associated with the presence of suicide clusters in previous studies (Sugg et al. 2023; Fontanella et al. 2018). We do not expect that our measures biased the results, but they do not allow us to fully explore this specific aspect of the neighborhood environment. Also, this study does not explicitly account for spatial and temporal effects, though we did include year, latitude, and longitude. The primarily null results found in this paper, as well as conflicting results in previous works on this topic, may be an artifact of the modifiable areal unit problem (Buzzelli 2020). Finally, this study of Detroit may not be generalizable to other metropolitan areas, especially those that differ with respect to race and economic trends.

## Conclusions

Place factors alone were not predictive of suicide or firearm suicide in our study of Detroit, Michigan at the census tract level, where variation in socioeconomic status, a commonly identified predictor, is limited. It may be that spatial measures characterizing the places where people live are less useful for explaining suicide risk in this setting. Future practice and study should consider focusing on both larger and smaller areas, including city and individual-level factors. For example, studies identifying features associated with suicide and firearm suicide risk might consider combining place-based variables with individual-level risk factors to understand their relative importance as well as the potential for interaction effects.

## Data Availability

The data used in this study are not openly availability due to sensitivity. Mortality data is owned and maintained by the Michigan Department of Health and Human Services.

## Appendix A: Machine learning algorithm tuning

**Table 3:** XGBoost Hyperparameter Tuning

Hyperparameter	Minimum	Maximum	FAS	Youth FAS	Suic	Youth Suic
eta	0	1	1	1	1	1
gamma	0.1	10	0.6180978	6.907296	9.868782	9.558025
max_depth	3	10	8	8	4	3
min_child_weight	1	10	7.18845	5.152555	7.217234	4.52785
subsample	0.5	1	0.8184902	0.5347971	0.807469	0.9781821
col_sample_by_tree	0.5	1	0.7306975	0.6210809	0.8108081	0.6670083
nrounds	100	6000	2269	4537	639	846

**Table 4:** Random Forest tuning

Outcome	Min	Max	Tuned mtry
FAS	5	13	12
Youth FAS	7	13	12
Suic	5	16	8
Youth Suic	5	16	10

## Appendix B: Exploratory analyses

**Table 5:** Summary statistics of covariates, comparing tract-years with and without firearm suicides

Variable	Sig	Tract-Years w/o FAS		Tract-Years w FAS	
		Median	Mean (SD)	Median	Mean (SD)
*Demographic Features*					
% Black		98.01	85.49 (27.10)	98.11	86.03 (26.39)
% Hispanic		0.95	6.54 (18.34)	0.94	6.78 (19.05)
% White		0.88	7.95 (14.92)	0.74	8.58 (15.68)
% Other race		0.81	5.77 (14.77)	0.87	5.38 (12.19)
% male aged 15 to 29		11.42	11.02 (2.79)	11.37	11.21 (2.41)
Residential racial segregation		− 0.97	− 0.78 (0.40)	− 0.97	− 0.77 (0.42)
Naturalized		16.00	34.72 (55.50)	17.00	37.40 (53.97)
Not US citizens		12.00	45.13 (93.18)	15.00	49.43 (95.53)
Population		2082.00	2194.80 (1058.25)	2416.00	2477.71 (951.65)
*Residential Stability*					
recent movers		82.00	83.56 (36.67)	77.00	81.54 (34.50)
% renters		47.57	49.63 (18.62)	44.70	47.02 (17.78)
Max # of arms-length transfers per parcel		2.00	2.07 (1.04)	2.00	2.28 (1.05)
% parcels w arms-length transfer (5 yrs)	*	0.03	0.03 (0.03)	0.03	0.04 (0.03)
*Socioeconomic Features*					
% female-headed households		16.65	16.56 (6.07)	16.37	16.62 (5.77)
% crowded households		14.00	16.35 (13.80)	15.00	16.76 (13.14)
% working in prof/bus/fin sectors		12.75	13.32 (7.38)	13.54	13.57 (5.92)
% adults high school graduates		69.07	69.23 (13.16)	73.65	71.57 (11.46)
Residential economic segregation		− 0.41	− 0.40 (0.20)	− 0.38	− 0.37 (0.18)
In armed forces currently		0.00	4.54 (17.87)	0.00	5.49 (19.70)
Median household income		28188.00	29885.94 (12678.68)	30375.00	32072.39 (12428.22)
% out of labor force		47.65	47.64 (10.40)	46.55	46.67 (7.45)
% below 150% of poverty line		44.53	42.66 (19.54)	36.97	38.35 (17.07)
% children aged 3+ in preschool		1.91	2.02 (1.06)	2.07	2.18 (1.12)
% children enrolled in K-12 private school		8.13	10.27 (8.19)	8.82	10.90 (7.70)
% public transit users		188.00	209.02 (143.50)	151.00	176.88 (113.76)
Unemployment rate		8.10	8.72 (4.75)	8.22	8.50 (3.64)
Median residential sales price (5 yrs)		25000.00	41214.71 (62935.29)	28500.00	44230.20 (42549.81)
% of parcels mortgage foreclosed		0.00	0.01 (0.01)	0.01	0.01 (0.01)
% of parcels tax delinquent		0.00	0.17 (0.18)	0.00	0.18 (0.20)
% of parcels tax foreclosed		0.00	0.02 (0.03)	0.00	0.02 (0.03)
*Physical features*					
Commercial buildings per sq. mi		93.12	99.12 (57.31)	91.37	100.34 (64.01)
Condos per sq. mi	*	1.96	14.74 (39.90)	2.27	22.96 (56.96)
Industrial buildings per sq. mi		2.92	8.45 (14.28)	2.33	7.38 (13.65)
Large (7+) apt. buildings per sq. mi		5.57	12.21 (19.41)	5.79	13.97 (26.48)
Small (1-6) apt. buildings per sq. mi		112.00	223.18 (292.58)	83.27	188.89 (249.43)
Mixed use buildings per sq. mi		0.00	4.41 (17.80)	0.00	4.75 (18.49)
Single family homes per sq. mi		1710.87	1579.57 (962.99)	2014.14	1806.57 (955.58)
Buildings per sq. mi. with grade D/F		73.22	98.34 (91.91)	55.28	82.40 (82.73)
Demolitions per sq. mi		0.00	2.01 (5.74)	0.00	2.18 (6.22)
% of parcels with vacant buildings		25.75	27.89 (14.61)	23.85	25.12 (11.59)
Building rehabilitations per sq. mi		0.00	1.35 (5.55)	0.00	1.67 (5.50)
Vacant lots per sq. mi	*	775.38	948.21 (719.90)	583.95	764.71 (610.97)
Protected land (acres)		1.63	18.36 (80.18)	2.41	10.56 (24.97)
Count of beer, wine, liquor stores		1.00	0.97 (1.07)	1.00	0.97 (1.20)
Count of drinking establishments		0.00	0.75 (1.55)	0.00	0.70 (1.41)
Federally qualified health center count		0.00	0.08 (0.35)	0.00	0.08 (0.30)
Hospital count		0.00	0.04 (0.36)	0.00	0.09 (0.63)
National Walkability Index		12.61	12.73 (1.73)	12.56	12.77 (1.53)
Protected land (percent)		0.63	3.89 (11.41)	0.78	3.16 (6.17)
Historic redline grade		3.00	3.09 (0.77)	3.00	3.01 (0.82)
Religious buldings (count)		3.00	3.09 (2.56)	3.00	3.42 (2.58)
Road network density		24.71	24.79 (5.22)	25.24	25.19 (5.01)
School count		0.00	0.68 (0.94)	0.00	0.72 (0.95)
Square miles		0.46	0.48 (0.27)	0.46	0.48 (0.23)
Distance to public transit (cat)		2.00	2.00 (0.59)	2.00	1.97 (0.59)
Longitude		− 83.11	− 83.10 (0.09)	− 83.12	− 83.11 (0.10)
Latitude		42.38	42.39 (0.04)	42.39	42.39 (0.04)

‘*’ denotes covariates that were significant at the 0.05 level in a generalized linear mixed effects logistic regression with a random intercept by census tract using only the single covariate and controlling for population

**Table 6:** Summary statistics of covariates, comparing tract-years with and without youth firearm suicides (YFS)

Variable	Sig	Tract-Years w/o YFS		Tract-Years with YFS	
		Median	Mean (SD)	Median	Mean (SD)
*Demographic Features*					
% Black		98.02	85.46 (27.12)	98.11	88.36 (23.62)
% Hispanic		0.95	6.61 (18.47)	1.00	4.73 (15.55)
% White		0.87	8.03 (15.02)	0.63	6.89 (13.32)
% Other race		0.81	5.77 (14.63)	0.73	4.76 (12.06)
% male aged 15 to 29		11.41	11.02 (2.77)	11.45	11.52 (2.01)
Residential racial segregation		− 0.97	− 0.77 (0.41)	− 0.97	− 0.81 (0.36)
Naturalized		16.00	34.92 (55.55)	18.00	35.98 (47.85)
Not US citizens		12.00	45.39 (93.31)	17.00	49.31 (96.60)
Population		2094.00	2206.47 (1053.43)	2785.00	2693.57 (896.56)
*Residential Stability*					
Recent movers		82.00	83.42 (36.52)	75.00	82.08 (35.51)
% renters		47.45	49.46 (18.58)	43.35	47.40 (17.60)
Max # of arms-length transfers per parcel		2.00	2.08 (1.04)	2.00	2.41 (1.16)
% parcels w arms-length transfer (5 yrs)		0.03	0.03 (0.03)	0.04	0.05 (0.03)
*Socioeconomic Features*					
% female-headed households		16.64	16.54 (6.06)	17.44	17.74 (5.65)
% crowded households		14.00	16.36 (13.79)	16.00	17.23 (12.19)
% working in prof/bus/fin sectors		12.84	13.35 (7.30)	12.72	12.93 (5.88)
% adults high school graduates		69.40	69.34 (13.06)	75.88	72.94 (11.61)
Residential economic segregation		− 0.41	− 0.40 (0.20)	− 0.37	− 0.37 (0.15)
In armed forces currently		0.00	4.62 (18.05)	0.00	4.77 (17.33)
Median household income		28305.00	30036.57 (12737.98)	30554.00	31445.66 (9709.63)
% out of labor force		47.61	47.58 (10.25)	45.96	46.63 (7.33)
% below 150% of poverty line		44.11	42.41 (19.41)	36.21	37.71 (17.34)
% children aged 3+ in preschool		1.91	2.03 (1.07)	2.21	2.22 (0.94)
% children enrolled in K-12 private school		8.19	10.32 (8.19)	9.01	10.71 (6.66)
% public transit users		187.00	207.35 (142.19)	141.00	165.43 (103.36)
Unemployment rate		8.13	8.71 (4.69)	7.84	8.35 (3.53)
Median residential sales price (5 yrs)		25350.00	41397.93 (61905.10)	27900.00	44296.25 (41245.07)
% of parcels mortgage foreclosed		0.01	0.01 (0.01)	0.00	0.01 (0.01)
% of parcels tax delinquent		0.00	0.17 (0.18)	0.18	0.21 (0.21)
% of parcels tax foreclosed		0.00	0.02 (0.03)	0.00	0.02 (0.03)
*Physical Features*					
Commercial buildings per sq. mi.		93.17	99.34 (57.79)	80.81	94.88 (62.27)
Condos per sq. mi.		1.96	15.30 (41.80)	4.06	20.97 (36.89)
Industrial buildings per sq. mi.		2.92	8.35 (14.19)	2.66	8.65 (15.68)
Large (7+) apt. buildings per sq. mi.		5.61	12.33 (20.06)	6.08	13.45 (22.24)
Small (1-6) apt. buildings per sq. mi.		106.97	220.89 (290.20)	102.53	196.16 (251.88)
Mixed use buildings per sq. mi.		0.00	4.41 (17.48)	0.00	5.23 (28.79)
Single family homes per sq. mi.		1723.76	1589.37 (963.28)	2154.01	1963.19 (937.63)
Buildings per sq. mi. with grade D/F		71.97	97.49 (91.35)	42.12	79.63 (87.17)
Demolitions per sq. mi.		0.00	2.01 (5.79)	0.00	2.56 (5.72)
% of parcels with vacant buildings		25.67	27.73 (14.46)	22.25	24.68 (11.52)
Building rehabilitations per sq. mi.		0.00	1.37 (5.53)	0.00	1.92 (6.10)
Vacant lots per square mile		768.59	936.79 (713.97)	509.06	770.74 (659.87)
Protected land (acres)		1.70	18.04 (78.02)	1.37	4.52 (9.48)
Count of beer, wine, liquor stores		1.00	0.97 (1.07)	1.00	1.05 (1.40)
Count of drinking establishments		0.00	0.75 (1.55)	0.00	0.61 (1.11)
Federally qualified health center count		0.00	0.08 (0.34)	0.00	0.08 (0.33)
Hospital count		0.00	0.04 (0.37)	0.00	0.13 (0.78)
National Walkability Index		12.61	12.74 (1.72)	12.58	12.55 (1.36)
Protected land (percent)		0.68	3.89 (11.20)	0.45	1.36 (2.38)
Historic redline grade		3.00	3.09 (0.77)	3.00	2.97 (0.81)
Religious buildings (count)		3.00	3.11 (2.57)	3.00	3.46 (2.50)
Road network density		24.72	24.80 (5.22)	25.43	25.69 (4.51)
School count		0.00	0.68 (0.94)	0.00	0.56 (0.72)
Square miles		0.46	0.48 (0.27)	0.46	0.45 (0.14)
Distance to public transit (cat)		2.00	1.99 (0.59)	2.00	1.95 (0.53)
Longitude		− 83.11	− 83.10 (0.09)	− 83.13	− 83.11 (0.10)
Latitude		42.39	42.39 (0.04)	42.39	42.39 (0.04)

‘*’ denotes covariates that were significant at the 0.05 level in a generalized linear mixed effects logistic regression with a random intercept by census tract using only the single covariate and controlling for population

**Table 7:** Moran’s I test for spatial autocorrelation for firearm suicide counts

Year	Firearm Suicide		Youth Firearm Suicide		Suicide		Youth Suicide	
	Moran’s I	p-value	Moran’s I	p-value	Moran’s I	p-value	Moran’s I	p-value
2012	0.0586	0.0322	− 0.0085	0.5619	0.0303	0.1599	0.0162	0.2794
2013	− 0.0189	0.6785	− 0.0250	0.7485	0.0335	0.1376	0.0489	0.0587
2014	0.0400	0.0975	0.0498	0.0420	− 0.0165	0.6506	0.0012	0.4447
2015	− 0.0265	0.7570	− 0.0155	0.6648	− 0.0291	0.7784	− 0.0220	0.7298
2016	0.0476	0.0638	− 0.0227	0.7282	0.0354	0.1259	− 0.0239	0.7324
2017	0.0353	0.1247	− 0.0280	0.7759	0.0123	0.3213	− 0.0117	0.5986
2018	− 0.0337	0.8162	− 0.0296	0.7904	− 0.0120	0.6000	− 0.0213	0.7038
2019	0.0025	0.4307	0.0260	0.1807	0.0392	0.1034	− 0.0053	0.5228

## Appendix C: Algorithm and model performance metrics for additional outcomes

**Table 8:** Model metrics: Youth Firearm Suicide

Model	AIC	AUC	Sens	Spec	AUPRC	Balanced Accur
Random Forest	–	0.6670	0.2500	0.8846	0.0495	0.5673
XGBoost	–	0.4342	0.1667	0.8825	0.0238	0.5246
Model 1, all vars	611.6	0.7511	0	1	0.0994	0.5000
Model 2, selected vars	560.9	0.7818	0	1	0.0799	0.5000
Model 3, offset, all vars	607.6	0.7540	0	1	0.1116	0.5000
Model 4, offset, selected vars	555.1	0.6624	0	1	0.0429	0.5000

**Table 9:** Model metrics: Suicide

Model	AIC	AUC	Sens	Spec	AUPRC	Balanced Accur
Random Forest	–	0.5902	0.2500	0.8505	0.1912	0.5502
XGBoost	–	0.5306	0.2639	0.7868	0.1996	0.5253
Model 1, all vars	1995.7	0.6514	0	1	0.2170	0.5000
Model 2, selected vars	1931.7	0.6330	0	1	0.2129	0.5000
Model 3, offset, all vars	1989.1	0.6483	0	1	0.2226	0.5000
Model 4, offset, selected vars	1927.9	0.6273	0	1	0.2139	0.5000

**Table 10:** Model metrics: Youth Suicide

Model	AIC	AUC	Sens	Spec	AUPRC	Balanced Accur
Random Forest	–	0.6861	0.4545	0.8472	0.0899	0.6509
XGBoost	–	0.6138	0.0909	0.9323	0.0606	0.5116
Model 1, all vars	928.1	0.7343	0	1	0.1223	0.5000
Model 2, selected vars	881.7	0.6833	0	1	0.0871	0.5000
Model 3, offset, all vars	925.3	0.7311	0	1	0.1262	0.5000
Model 4, offset, selected vars	883.6	0.6762	0	1	0.0861	0.5000

## Abbreviations

AUC: Area under ROC curve
AUPRC: Area under precision-recall curve
GLMM: Generalized linear mixed model
ICD: International classification of diseases
ICE: Index of concentrations at the extremes
ROC: Receiver operating characteristic curve
XGBoost: Extreme gradient boosting
